# Insight into Structural Characteristics of Protein-Substrate Interaction in Pimaricin Thioesterase

**DOI:** 10.3390/ijms20040877

**Published:** 2019-02-18

**Authors:** Shuobing Fan, Rufan Wang, Chen Li, Linquan Bai, Yi-Lei Zhao, Ting Shi

**Affiliations:** State Key Laboratory of Microbial Metabolism, Joint International Research Laboratory of Metabolic and Developmental Sciences, School of Life Sciences and Biotechnology, Shanghai Jiao Tong University, Shanghai 200240, China; bigyellowjar@sjtu.edu.cn (S.F.); wangrufan@sjtu.edu.cn (R.W.); l1105309790@sjtu.edu.cn (C.L.); bailq@sjtu.edu.cn (L.B.); yileizhao@sjtu.edu.cn (Y.-L.Z.)

**Keywords:** pimaricin thioesterase, protein-substrate interaction, macrocyclization, molecular dynamics (MD) simulation, pre-reaction state

## Abstract

As a polyene antibiotic of great pharmaceutical significance, pimaricin has been extensively studied to enhance its productivity and effectiveness. In our previous studies, pre-reaction state (PRS) has been validated as one of the significant conformational categories before macrocyclization, and is critical to mutual recognition and catalytic preparation in thioesterase (TE)-catalyzed systems. In our study, molecular dynamics (MD) simulations were conducted on pimaricin TE-polyketide complex and PRS, as well as pre-organization state (POS), a molecular conformation possessing a pivotal intra-molecular hydrogen bond, were detected. Conformational transition between POS and PRS was observed in one of the simulations, and POS was calculated to be energetically more stable than PRS by 4.58 kcal/mol. The structural characteristics of PRS and POS-based hydrogen-bonding, and hydrophobic interactions were uncovered, and additional simulations were carried out to rationalize the functions of several key residues (Q29, M210, and R186). Binding energies, obtained from MM/PBSA calculations, were further decomposed to residues, in order to reveal their roles in product release. Our study advanced a comprehensive understanding of pimaricin TE-catalyzed macrocyclization from the perspectives of conformational change, protein-polyketide recognition, and product release, and provided potential residues for rational modification of pimaricin TE.

## 1. Introduction

Produced predominantly by the genus *Streptomyces*, polyene polyketides consist of a large family of natural compounds [1,2], including pimaricin [3], amphotericin B [4], nystatin A1 [5], and candicidin/FR008 [6]. The members of this class are widely used in clinical medicine for their broad spectral antifungal properties [7]. With constant progress in scientific research, their potential pharmaceutical values on antiviral, antiprotozoal, antiprion, and anticancer activity have been progressively clarified [8,9].

As a potent polyene antibiotic produced by *Streptomyces natalensis*, pimaricin (i.e. natamycin) primarily functions in the treatment of fungal infections caused by *Candida*, *Fusarium*, *Penicillium*, and *Aspergillus* organisms [10]. It is also known as an additive in food industry [11]. Pimaricin was approved by the Food and Drug Administration (FDA) as a drug for fungal keratitis in 1978 [12]. Ergosterol constitutes a major component in fungal and trypanosomatids plasma membrane, while absent in animal cells [13]. Pimaricin serves to bind specifically to ergosterol, downregulate vesicle trafficking, suppress membrane protein transport, and interfere with endocytosis, as well as exocytosis without permeabilizing the membrane [14,15,16]. Its strong performance in clinical trials makes pimaricin appealing to researchers, and its biosynthetic pathway modification and drug design have become new science hotspots [17].

Pimaricin is synthesized by type I polyketide synthases (PKSs), which consists of several covalently-connected multi-domain “modules.” Each module contains a set pattern of domains, with acyltransferase (AT) adding acyl-CoA building blocks, acyl carrier protein (ACP) carrying the polyketide between modules, and ketosynthase (KS) accepting the growing chain from ACP [18]. An extra combination of domains, such as ketoreductase (KR), dehydratase (DH), methyltransferase (MT) were responsible for the production of distinctive macrolactones [19,20,21]. Situated in the last module, the thioesterase (TE) domain off-loads the ACP-tethered polyketide from PKS via macrocyclization or hydrolysis.

Consistent with serine hydrolase, a two-step mechanism has been proposed for TE-mediated catalysis of macrocyclic polyketides [22]. The first step is acylation of a universally conserved serine residue in the catalytic triad, generating an acyl-enzyme intermediate and stabilized for a considerable time [23]. The second step takes place with an intra-molecular hydroxyl group on polyketide which initiates a nucleophilic attack and leads to cyclization, or hydrolysis of the acyl-enzyme intermediate with no efficient intra-molecular nucleophile present.

**Figure 1:**
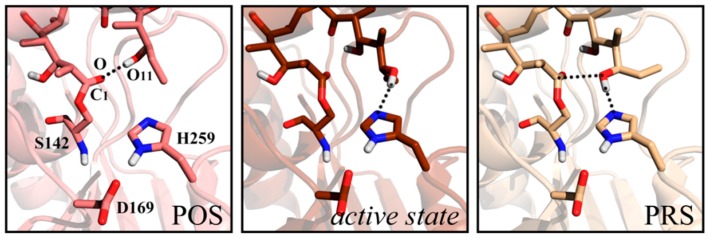
Structures of pre-organization state (POS), active state and the pre-reaction state (PRS) of 6-deoxyerythronolide B synthase (DEBS) thioesterase (TE) system.

In our previous work concerning 6-deoxyerythronolide B synthase (DEBS)-TE [24], a hydrogen bond emerged between the polyketide chain terminal hydroxyl group O_11_ and carbonyl oxygen O (Figure 1), as accompanied by the swing of C_11_ ethyl of substrate. This structure has been reported in Trauger’s work in 2001, where it was referred to as the “pre-organization state” (POS). According to Trauger [25], the “product-like” conformation might contribute to pre-organization of the substrate for cyclization. The conformation with a hydrogen bond, forming between the O_11_ and Nε of His259 in the catalytic triad, was defined as an *active state* in our study. This conformation maintained for ~100 ns in our simulations with considerable steadiness. However, the distance of O_11_-C_1_ for nucleophilic attack was larger than 6 Å in *active state*. Finally, an advantageous conformer (the pre-reaction state, PRS) was found [24] after ~270 ns MD simulation, which possessed both hydrogen bond O_11_-Nε_H259_ and an appropriate distance between O_11_ and C_l_ to facilitate nucleophilic attack. Critical to mutual recognition and catalytic preparation between TE and substrate, the PRS seemed decisive in the occurrence of macrocyclization.

To understand the molecular basis of pimaricin-TE (pima-TE) catalyzed macrocyclization, MD simulations were employed on enzyme-substrate complex. POS, *active state*, and PRS were discovered during 5 × 50 ns molecular dynamics (MD) simulations, and the conformational transition between POS and PRS was explored. The structural characteristics of POS and PRS were uncovered by conducting analyses of hydrogen-bonding and hydrophobic interactions. Additional simulations on several mutants (including Q29A, M210G, R186F, R186Y and S138C) were carried out to validate the functions of several key residues in substrate recognition and product release. At last, the binding energies of enzyme-product complex were obtained through MM/PBSA calculations, and with critical residues highlighted. We also provided an explanation on the departure of product from the active site.

## 2. Results and Discussion

### 2.1. Key Structural Conformations in MD Simulations

**Figure 2:**
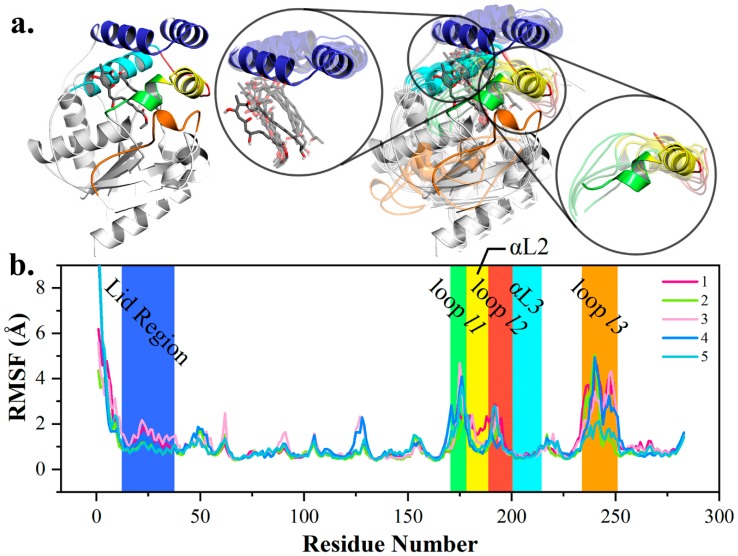
Conformational change of pima-TE system during molecular dynamics (MD) simulations. (**a**) Structural variations between post (opaque) and pre-simulation (transparent) complexes, with lid region, polyketide chain, α-helix αL2, αL3 and loop *l1*, *l2* & *l3* colored in tv_blue, gray, yellow, cyan, green, red and orange. (**b**) Root-mean-squared fluctuation (RMSF) of five trajectories with key structural elements highlighted.

Intrigued by the recognition mechanism of pima-TE, 5 × 50 ns MD simulations were performed on constructed complex. Root-mean-square deviation (RMSD) analysis revealed that all five trajectories attained equilibrium (Figure S1). The root-mean-squared fluctuation (RMSF) values highlighted six parts on pima-TE. Firstly, the lid region was violently jacked up by the erected polyketide (Figure 2). As a polyene molecule with 26-atom skeleton, pimaricin’s accommodation would require a larger space, compared with pikromycin, a 14-membered ring. It was conceivable that the relaxation of the substrate would incur close contact with the lid. Next, as components of the channel, αL2, as well as loop *l1* & *l2*, presented evident structural dynamism and various coiling states, while αL3 exhibited negligible fluctuation. Helix αL2 was proposed to wield a larger influence on protein-intermediate recognition than αL3, and *l1* and *l2* were responsible for the exit and entrance size. At last, RMSF indicated that loop *l3* adopted larger fluctuations than loop *l1* and *l2*, and the b-factor calculation [26,27] disclosed an inherent mobility of loop *l3*.

**Figure 3:**
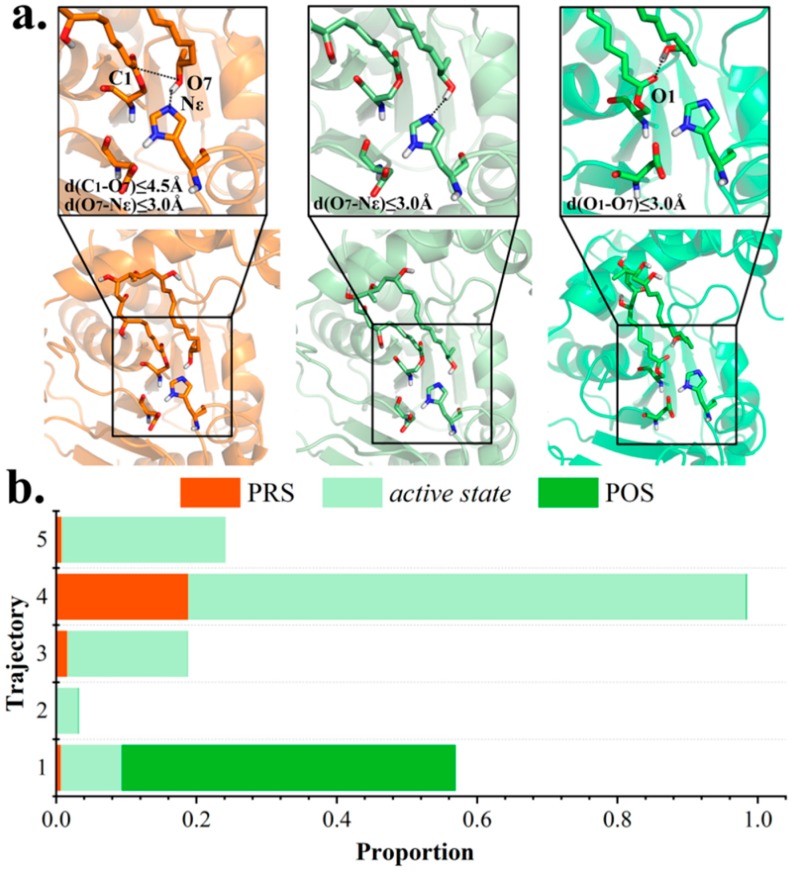
Classification of trajectory frames based on polyketide chain conformation. (**a**) Representative structures of PRS, *active state* and POS extracted from clustering analysis. (**b**) Proportion of PRS, *active state*, and POS in each trajectory.

Next, conformational variations at active site in each trajectory were carefully studied. The entire 250 ns trajectory was divided into three categories, based on distance measurement. With a hydrogen bond coming into being between terminal hydroxyl O_7_ and Nε_H261_ (distance (O_7_-Nε_H261_) ≤ 3.0 Å), the intermediate was regarded ready to be de-protonated by H261, namely, an *active state*. The *active state* was observed in all five trajectories (8.7, 3.1, 17.1, 79.5, and 23.4%, respectively), with the highest proportion in md4 (Figure 3). Moreover, the terminal O_7_ was proposed to be conducive for nucleophilic attack onto carboxyl C_1_ with distance (O_7_-C_1_) ≤ 4.5 Å. The PRS was defined as both criteria were met, and was present in md4 for 4700 frames (18.8%, Figure S2); in other trajectories, PRS appeared with a significantly lower frequency, testifying to its unsteadiness as a transient state.

Distinguished from PRS and *active state* that ultimately lead to macrocyclization, inactive conformations are susceptible to hydrolysis. Notably, among inactive conformations, the POS, which is characterized by a hydrogen bond between O_7_ and carbonyl oxygen O_1_ of substrate, was also observed within md1 for 11896 frames (47.6%, Figure S2), whereas it was nearly absent in others (3, 2, 20 and 0 frames in md2–5).

### 2.2. Conformational Transition between POS and PRS

Next, the transformation between POS and PRS was studied using dihedral angle C_α_-C_β_-C_γ_-O_7_ as an indicator of polyketide terminal rotation. In PRS, bond C_α_-C_β_ ran anti-parallel against C_γ_-O_7_ (−180°), but in POS, the dihedral angle was altered to an acute angle fluctuating between (−30°, −70°).

**Figure 4:**
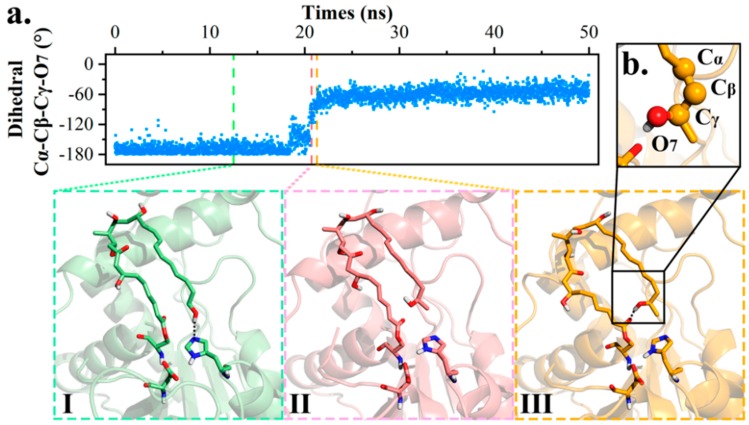
Conformational transformation between PRS and POS. (**a**) Dihedral value representation of md1 along with intermediate conformation change. (**b**) Presentation of dihedral angle C_α_-C_β_-C_γ_-O_7_.

The conformational flip took place in 18–22 ns of md1 trajectory, with conformation altering progressively from PRS (−180°) to POS (−60°). As presented in Figure 4, terminal hydroxyl O_7_ firstly swung up and disassociated from H261, followed by C_β_-C_γ_ twisting clockwise and terminal methyl oriented towards the entrance (I→II). Further, the intermediate swelled to diminish distance O_7_-O_1_. After quick adjustment, POS came into being and maintained for rest of the trajectory (II→III).

An energy calculation was also conducted to investigate the structural stability of aforementioned conformations. As expected, POS harbored a lower energy than PRS by 4.58 kcal/mol, indicating the steadiness of the O_7_-O_1_ intra-molecular hydrogen bond. On the other hand, the *active state* was calculated to be 0.18 kcal/mol less stable than PRS. The slight difference prompted that conformational transition between *active state* and PRS would easily achieve through C_β_-C_γ_ bond rotation.

In conclusion, a conformational transformation between POS and PRS was accomplished through dihedral flip and conformation adjustment, and the energies on POS, *active state*, and PRS were computed to understand the reaction process.

### 2.3. Hydrophilic and Hydrophobic Interactions in Pima-TE System

**Figure 5:**
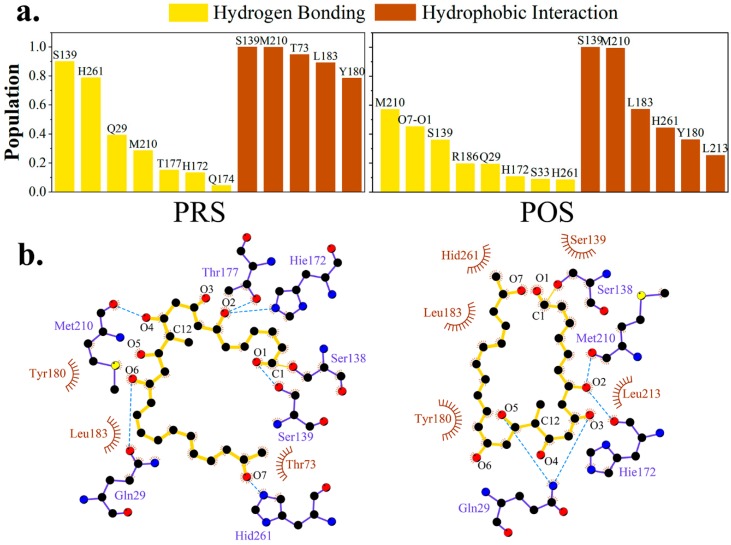
Hydrogen-bonding and hydrophobic interaction network variations in PRS and POS. (**a**) Proportion of top-ranked hydrogen bonding interactions. (**b**) Diagram of protein-substrate interaction produced by ligplot^+^ [28]. Backbone of the polyketide substrate was colored in yellow, and residues providing hydrogen bonding and hydrophobic interactions in slate_blue and brown.

Based on MD simulations, hydrogen bonding and hydrophobic interactions between pima-TE and substrate were studied. As exhibited in Figure 5, in PRS, loop *l1* (residue 170–177) played a crucial part in fastening the substrate. The atoms O_2_, O_3_, O_4_ or O_5_ were anchored by H172 (13.35%), T177 (15.20%) and Q174 (4.55%) without fixed pattern. Residue Q29, stretching downward from the lid region, served as a crane to lift up O_6_ and gave rise to an erected molecule (39.26%). The main chain of M210 fixed O_4_ at the center of the molecule (28.56%), while its side chain laid parallel to the hydrophobic area of conjugated polyene (99.92%). The hydrophobic segments of T73, L183 and Y180 were also oriented towards the polyketide chain and worked jointly to conserve a water-free sub-environment with frequencies of 94.88, 89.27 and 78.50%, respectively.

Under the circumstance of POS, Q29 (19.38%) and H172 (10.71%) assisted the intermediate erection, except for this time the molecule slighted twisted to stabilize the intra-molecular hydrogen bond, which enabled S33 from the lid region to contribute in the hydrogen bonding network (8.90%). Identical to PRS, M210 again assumed the role of both a hydrophilic stake (56.98%) and a hydrophobic driving force (99.28%). Besides Y180 (36.17%) and L183 (57.06%) as in PRS, L213 also participated in hydrophobicity maintenance (25.43%).

In a word, binding modes of pimaricin polyketide with TE shared considerable similarity between PRS and POS, with Q29, M210 and residues on loop *l1* interacting with the chain via hydrogen bonding, and M210, Y180, and L183 contributing to hydrophobic network.

### 2.4. Key Residues Analyzed Via Mutant Simulations

#### 2.4.1. Mutation 1-Q29A

**Figure 6:**
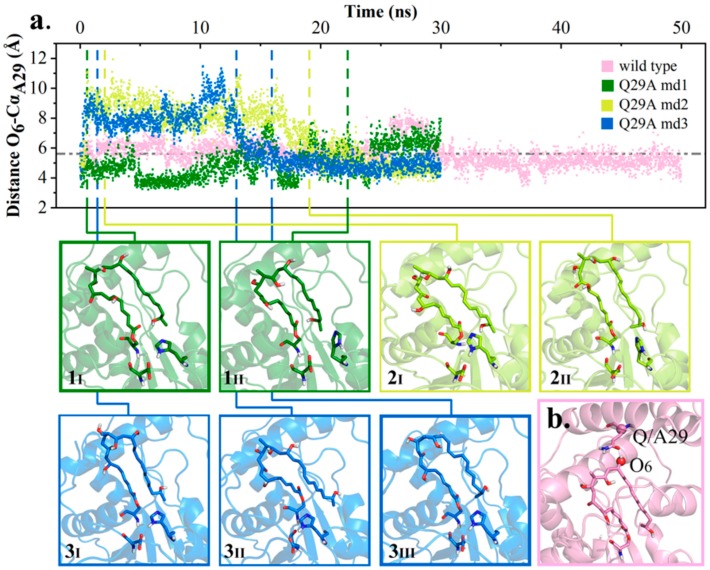
Instability of polyketide conformation. (**a**) Distance O_6_-C_Q/A29_ in wild type md4 and Q29A md1–3 along with conformation transformation. (**b**) Diagram of distance O_6_-C_Q/A29._

According to the analyses of wild type simulations, Q29, located at lid region of pima-TE, could mediate the distance O_7_-Nε_H261_ within a favorable range through bonding with O_6_ of substrate. When mutated to Ala, with Q29’s side chain shortened and hydrogen bond abolished, it was speculated that the substrate would fall off from its original position. Here, the distance between O_6_ and Cα of Q/A29 (designated as O_6_-C_Q/A29_) was utilized to depict the substrate’s spatial displacement. As shown in Figure 6, the distance O_6_-C_Q/A29_ fluctuated acutely in Q29A trajectory md2 and md3, with the substrate either overlength (**2_I_**, **3_I_**), hydrogen bonding to other residues (**3_II_**), or drifting aimlessly (**2_II_**, **3_III_**). The erratic change also decreased PRS formation by a large margin (4.37% vs. 0.54%, Table S1). On the other hand, POS was observed in Q29A md1 with a frequency of 80.84% (**1_I_**, **1_II_**).

From our perspective, Q29 could regulate hydrogen bond O_7_-Nε_H261_ and PRS formation by binding the substrate position with a hydrogen bond, while having little effect on POS.

#### 2.4.2. Mutation 2-M210G

**Figure 7:**
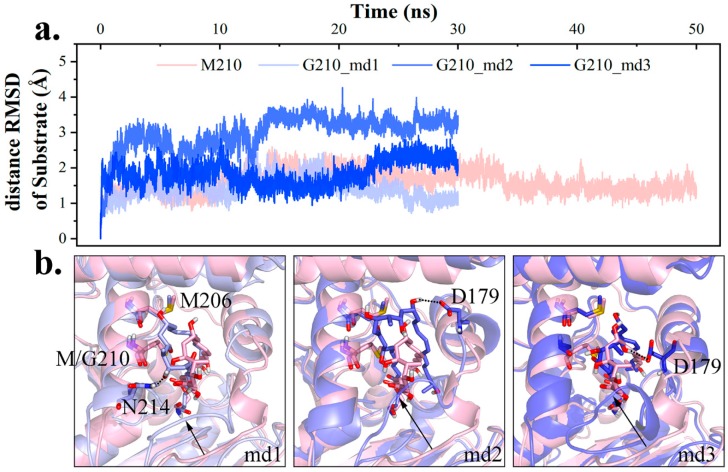
Conformational change of intermediate change upon M210 mutation. (**a**) Distance RMSD (dRMSD) value of substrate backbone in wild type md4 and M210G (lightpink for wild type, lightblue, slate and tv_blue for M210G md1–3). (**b**) Diagram of the dominant structure in each trajectory.

To validate M210’s function in hydrophobic interaction network, M210 was mutated into Gly. The substrate backbone’s distance RMSD (dRMSD) was calculated with the first frame as a reference. As seen in Figure 7, the larger dRMSD signified variation in substrate conformation, and its irregularity suggested volatility. Furthermore, new patterns of hydrogen bonding were observed in mutant (Figure 7): In md1, the polyketide chain leaned towards αL3 and interacted with N214 (23.08%); in md2 and md3, the substrate slightly rotated and bonded with D179 on αL2 (76.35% and 87.10%). Having lost M210 as a hydrophobic barrier, the polyketide chain would adjust its position, and M206 from the neighboring cycle of αL3 exhibited hydrophobicity. Owing to the altered interaction network, it was hard for the substrate to attain PRS as in wild type complex (4.37% vs. 0.72%).

Specifically, the aforementioned conformational change of substrate also produced a similar effect on loop *l1* seeking hydrogen bonding, and gave rise to a shrinking channel exit, while a bigger exit would be favored in product release. Taken together, M210 was crucial in maintaining the polyketide chain in between αL2 and αL3, which was conduced to protein-substrate interaction and an advantageous channel exit shape.

#### 2.4.3. Mutation 3-R186F & R186Y

**Figure 8:**
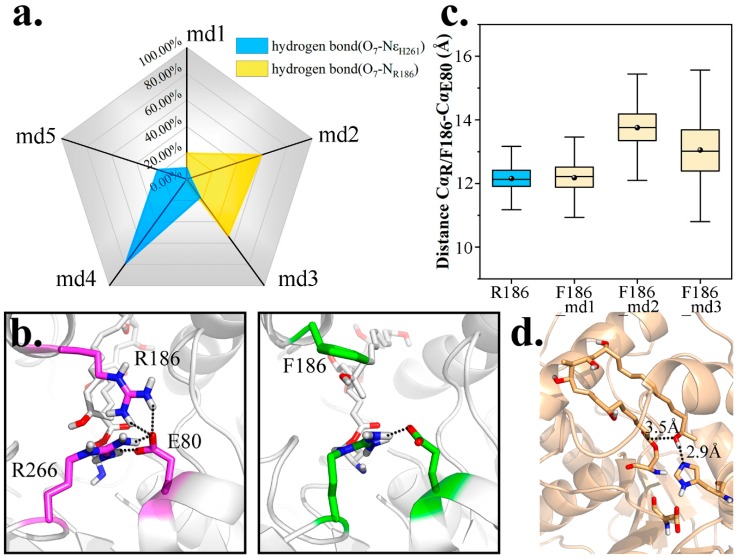
Mutational trials on R186. (**a**) Radar chart indicating the proportion of hydrogen bond O_7_-Nε_H261_ and O_7_-N_R186_ formation within 5 wild type simulations. (**b**) Larger entrance of pima-TE after R186F mutation. (**c**) Coupling and non-coupling states of three entrance residues (R/F186, E80 and R266) located on different structure elements. (**d**) A favorable PRS emerged in R186Y md3.

As a residue containing multiple hydrophilic groups, R186 bonded with O_7_ for a rather high probability in wild type complex simulations. As seen in Figure 8a, in md1–3, high frequency of O_7_-N_R186_ bonding could account for the scarce existence of O_7_-Nε_H261_ interaction. To promote PRS formation, R186 was firstly mutated to Phe.

To our disappointment, the frequency of PRS formation did not improve (4.37% vs. 1.64%). It was determined that E80 was coupled with R186 and R266, to pose a spatial barrier at the entrance and prevent the admission of other substrate, while functionally maintaining the closure and hydrophobicity of substrate pocket. Nevertheless, when R186 was mutated to Phe, a crack appeared (Figure 8b), and frequency of E80-R266 interaction was lowered as well (Table S2). Worse still, lacking the tying force, the distance Cα_F186_-Cα_E80_ also increased, implying a larger entrance (Figure 8c).

Based on our findings, pima-TE was re-modified into R186Y mutant. This time, we endowed a hydroxyl to the side chain of mutated residue to bond with E80, while the remainder stayed hydrophobic. We were more than pleased to find a significant rise in PRS ratio (4.37% vs. 18.14%), with Y186-E80 bonding partly restored (Table S3). Of particular note, a close-to-reaction PRS conformation appeared in md3 and maintained for over 10 ns, with the terminal methyl oriented towards the entrance and forcing O_7_ closer to C_1_ (Figure 8d). We thus regard R186Y as a promising modification towards pimaricin productivity advancement.

#### 2.4.4. Mutation 4-S138C

**Figure 9:**
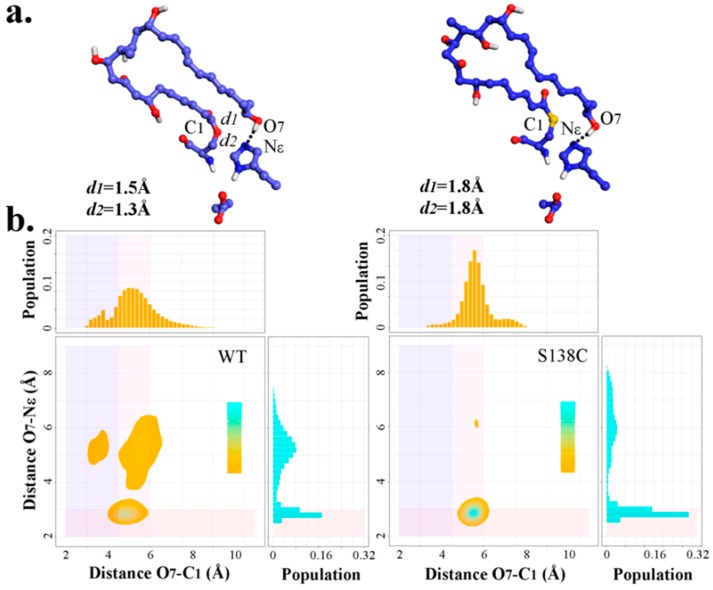
(**a**) Diagram of representative PRS conformations in wild type (**left**) and S138C (**right**) trajectories. (**b**) Density map and marginal histogram indicating the distribution of all frames on the basis of distances O_7_-Nε_H261_ and O_7_-C_1_ in wild type and S138C trajectories. The rectangles in light-coral, slate-blue, and thistle highlight points with distance (O_7_-Nε_H261_) ≤ 3.0 Å, distance (O_7_-C_1_) ≤ 4.5 Å and 4.5 Å ≤ distance (O_7_-C_1_) ≤ 6.0 Å, respectively.

As presented by Koch et al. [29], compared with pikromycin synthase (PICS)-TE_WT_, PICS-TE_S148C_ could promote macrocyclization efficiency by over 300%. Therefore, pima-TE_S138C_ mutant were subjected to MD simulations to study whether the superiority of Cys over Ser applied in pima-TE as well. After the clustering analysis, the dominant polyketide structure of each S138C trajectory demonstrated unbelievable similarity (Figure S3). As seen in Figure 9, S138C frames were significantly more concentrated in the conducive range for reaction compared to wild type ones, suggesting potential catalytic advantage. However, due to O→S atomic radius enlargement, bond length involving O/S increased by 0.3 Å, and 0.5 Å, respectively, and distance O_7_-C_1_ would mostly gather around 5.5 Å in mutated complex. The density of advantageous conformations in S138C system strongly suggested the favorability of this mutation.

### 2.5. Study on TE’s Effect on the Release of Pimaricin Product

**Figure 10:**
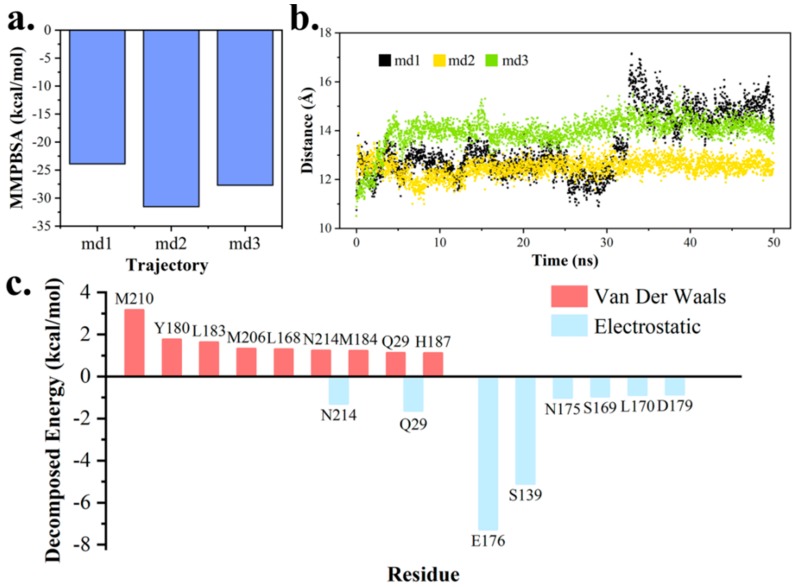
MM/PBSA analysis on the outward trend of macro-lactone product. (**a**) Binding energy between pima-TE and product ring. (**b**) Distance between ring mass center and Cα_S138_. (**c**) Residues with top-ranked van der Waals (VDW) and electrostatic contributions to binding free energy.

The binding energy between pima-TE and polyketide product was calculated with MM/PBSA program (Figure 10a). In study of product (i.e., MOL) movement across the channel, distance between its mass center and Cα_S138_ was measured (Figure 10b). As a result, the product migrated towards the exit for approximately 4 Å in md1, while it hardly moved in others. Therefore, md1 was regarded to have a tendency of product release, and the other two disclosed the stabilization effect produced by the protein. Energy decomposition revealed residues around the exit to play key parts in protein-product interactions (Figure 10c), and to assume important roles in product release. (Figure S4)

**Figure 11:**
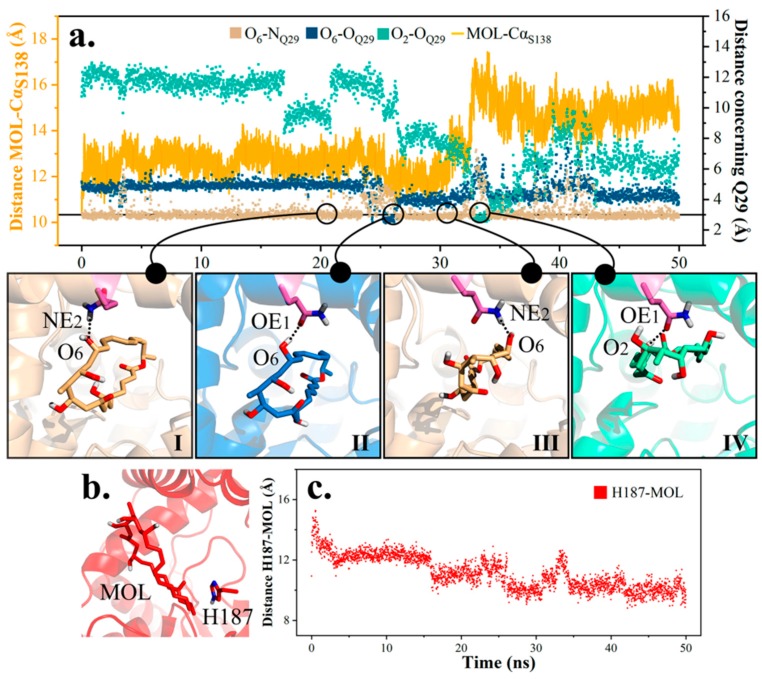
Diagram of product movement in substrate channel in md1. (**a**) Three hydrogen bonding modes between Q29 and MOL and their transformation. (**b**) Spatial location of H187 with respect to MOL. (**c**) Distance H187-MOL in md1.

Next, a careful analysis was conducted on the disengagement of product in md1, and three patterns of hydrogen bonding between Q29 and product were generalized (Figure 11). For the first 25 ns, the product remained its original state and O_6_ from the product ring continued bonding to Q29 (I). Afterwards, in cooperation with Q29’s side chain turnover (II), the ring lied down a little and interacted with Q29 from the right (III). Then, the free hydroxyl on Q29 (OE_1_) grasped O_2_ from the other side of the molecule, further enabling the molecule to lie flat (IV). In a word, steps II-III and III-IV played decisive roles in altering the product’s layout and pulling the product farther away from the active site. Due to distinguished distribution of hydrophilic and hydrophobic areas on protein, rotation of the product might partly attenuate its interaction with peripheral residues, and impel the product’s departure.

On the other hand, H187 seemed to provide thrust towards the release of product (Figure 11). Distance between the mass center of H187 and product ring shrank along the simulation, revealing established hydrophobic interaction between the imidazole of H187 and terminal methyl on the product (Figure 11).

Taken together, after cyclization, the product would stay in the vicinity of active site for a while due to van der Waals (VDW) and electrostatic interactions from peripheral residues. Later on, the product layout was altered by molecule rotation, varied hydrogen bonding, etc., which impaired the spatial constraint, and caused the ring to gradually migrate towards the exit, with Q29 hydrogen bonding as a driving force and H187 as a rear helper.

## 3. Discussion

Due to the limitation of experimental instruments, present computational strategies combining homology modeling, molecular docking, MD simulation, and QM/MM calculation have been extensively utilized to provide insight into atomistic details in protein-substrate recognition and catalytic mechanism. Over recent years, packages and software [30,31,32] to study protein-substrate interaction have sprung up relentlessly, and MD simulation has become a regular routine herein [33,34,35].

In this work, MD simulations were carried out on pima-TE-substrate/product complexes. Residues playing critical roles in product recognition, assembly, and release were uncovered through hydrogen bonding and hydrophobic interaction network analysis, which could be obtained from representative conformations of trajectories, as well as decomposition of MM/PBSA binding energy. Q29 and M210 might contribute to tight binding effect, and the structural correlation between protein and substrate was reduced once they were eliminated. R186 was uncovered to maintain pocket hydrophobicity yet distract the substrate from a proper position, and its mutation to Tyr could benefit macrocyclization by raising the proportion of advantageous conformation. The computer-aided methods could provide theoretical basis to enzyme clarification.

Since the transition states of enzyme catalysis were hard to obtain in silico, we chose pre-reaction state (PRS) as an evaluation indicator. According to our previous research [26,36], PRS was the very prior stage of macrocyclization in terms of both structure and energy, and its formation was decisive to TE cyclization. The proportion of PRS was regarded as the degree of reaction readiness. Besides, PRS proportion was used in mutated systems as well to help elucidate the functions of these residues and speculate their effect on TE activity. However, a more accurate account of the mutation required explanation in energy and experimental verification as well.

To conclude, the study approach applied in our work involved protein-substrate interaction, residue targeting, and mutation analyses with PRS occurrence as an indicator. The strategy could provide structural rationale for TE-substrate complex and guide future experiments on design of efficient protein mutants or novel compounds.

## 4. Materials and Methods

### 4.1. System Preparation

Given the unavailability of pima-TE crystallization data from Protein Data Bank (PDB), initial structure of pima-TE was produced through homology modeling with PICS-TE [37] (PDB: 2H7Y) as a template (sequence similarity: 48.1%). Twenty pima-TE models were generated in the discovery studio 3.5 [38]. The one with the lowest total energy was selected, and its stereochemical quality was further validated by Procheck [39], with 93.7% of its residues falling in the most favored region.

Considering the extensively-acknowledged catalytic process of pimaricin PKS, the mature pimaricin product was disconnected at carbonyl C_1_, and the lactonic ring as well as exocyclic mycosamine were removed. Furthermore, carboxyl on C_12_ was also substituted by a methyl. The precursor was optimized with Gaussian09 [40] AM1 method [41], after which the buckled conformation still sustained. The energetically-stabilized substrate was then covalently bonded to active site Ser138 on pima-TE model, with a hydrogen bond forming between its terminal hydroxyl and Nε of active site His261. Protonation state of His261 was altered to HID to facilitate PRS formation. The polyketide-bound acyl-enzyme intermediate was utilized as the initial structure of MD simulations.

During the preparation of the system parameters, an N-terminal cap (-CO-CH_3_) and a C-terminal cap (-NH-CH_3_) were firstly added onto the Ser138 to block its ends. Conformational optimization at the level of HF/6-31G(d) was then employed on the intermediate, and its electrostatic surface potential (ESP) charge was computed. Afterwards, a two-step restrained electrostatic potential (RESP) model was applied to determine charge distribution on the substrate. Finally, two prior-added caps were removed, and parameters for the intermediate were generated by the Antechamber package, on the basis of which topology files for protein-substrate complexes were prepared with *tleap* module in AMBER 14. Through *tleap*, pima TE-substrate system was placed in an octahedral TIP3P water box [42], with 12 sodium ions added to maintain charge neutralization.

### 4.2. Molecular Dynamics Simulation

Starting from the solvated polyketide-bound acyl-enzyme intermediate, classical molecular dynamics simulations were carried out utilizing AMBER14 [43] ff03.r1 force field [44]. The system was firstly subjected to 10,000 steps of steepest descent energy minimization followed by 1000 cycles of conjugate gradient minimization with bonds involving hydrogen constrained by SHAKE algorithm [45], and then another 10,000 steps of steepest descent energy minimization followed by 5000 cycles of conjugate gradient minimization with no constraint exerted. The system was then gradually heated from 0 to 300 K through 25000 iterations. After a 200ps-equilibrium in NPT ensemble, five 50-ns simulations (300 K, 1 atm) with different random seeds were conducted. The VDW interactions were cut off at 10 Å and long-range electrostatic interactions were calculated with particle mesh Ewald (PME) method [46]. Analyses of trajectories were performed using *cpptraj* in Ambertools14.

### 4.3. Quantum Mechanics/Molecular Mechanics) Calculation

Quantum mechanics/molecular mechanics (QM/MM) calculations were performed with a two-layered ONIOM method [47,48] in Gaussian09 program. Geometrical snapshots from the dominant MD cluster were extracted as PRS, *active state*, and POS, and were further subject to geometry optimization. The quantum mechanical (QM) layer consisted of side chains of active site triad (Ser138, Asp166 and His261) and the polyketide chain, which added up to 96 atoms and bore one negative charge. The optimization process was carried out under M06-2X [49] functional and basis set 6-31G(d) [50].

### 4.4. Simulation of Site Mutation Proteins

Based on the analyses, M210 and Q29 were selected as key residues in protein-substrate interaction. Considering the optimization of pima-TE, R186 was mutated to Phe and Tyr in succession to reduce its interference against PRS formation. In accordance with a previously published article of Koch et al. [29], S138 was also mutated to Cys to examine pima-TE_S138C_’s effectiveness. Single site mutation was employed directly on the initial structure of wild type pima-TE, and all mutants (M210G, Q29A, R186F, R186Y, S138C) went through 30–50 ns simulations following identical procedures as mentioned in Section 4.2.

### 4.5. Free Energy Calculation and Conformational Stability Analysis

The polyketide chain, which was extracted from dominant structure in wild type md4, was manually rang up and subjected to conformational optimization with Gaussian 09 AM1 method. The optimized product was then docked into the channel with C_1_ adjacent to the active site. After the model construction, 3 × 50 ns MD simulations was carried out with AMBER14 program.

After clustering analysis in *cpptraj*, a 20 ns segment with dominant conformation was extracted from each trajectory, and was further subject to a molecular mechanics Poisson-Boltzmann surface area [51] (MM/PBSA) calculation to estimate the free energy difference (Δ*G*^tot^) between bound and detached states of product-protein complexes in solution. The MMPBSA.py program in AMBER14 was performed, and the free energy discrepancy was decomposed to peripheral residues in terms of hydrophobic and electrostatic forces. Table 1 lists the number and duration of all MD simulations utilized in the study.

## 5. Conclusions

In this paper, MD simulations were utilized as a primary tool to explore pimaricin TE catalysis on an atomic level. Firstly, 5 × 50 ns trajectories on polyketide were conducted in search of pre-reaction states (PRS), and transformation between POS and PRS were examined. POS was found to bear lower energy, yet less mature conformation in comparison with PRS. Protein-polyketide hydrogen bonding and hydrophobic interactions were deciphered, with several key residues subjected to mutations. As discovered, Q29 was responsible for holding a polyketide hydroxyl and controlling the substrate position, and M210 contributed to favorable protein-ligand interaction by virtue of its hydrophobicity. R186Y might promote productivity by reducing the interference on PRS formation, and S138C could effectively enhance the proportion of required conformations. Ultimately, the MM/PBSA program was employed to unveil residues mediating product release, and the postulation of a mechanism of polyketide product departure from the active site was proposed. We gave a comprehensive overview on pima-TE catalysis, with computational methods, and offered opinions for protein engineering.

## Figures and Tables

**Table 1:** List of MD Runs Performed.

Substrate Type	Name	System	No. of Runs Per Complex	Length Per Run (ns)
Polyketide Chain	wild type	pima-TE_WT_ + polyketide chain	5	50
	M210G	pima-TE_M210G_ + polyketide chain	3	30
	Q29A	pima-TE_Q29A_ + polyketide chain	3	30
	R186F	pima-TE_R186F_ + polyketide chain	3	30
	R186Y	pima-TE_R186Y_ + polyketide chain	3	30
	S138C	pima-TE_S138C_ + polyketide chain	3	50
Product	ring	pima-TE_WT_ + product	3	50

## Supplementary Materials

Supplementary materials can be found at http://www.mdpi.com/1422-0067/20/4/877/s1.

## Abbreviations

PKS	Polyketide Synthase
TE	Thioesterase
MD	Molecular Dynamics
PRS	Pre-reaction State
POS	Pre-organization State

## References

[1] Gil J.A., Martin J.F., W. Strohl M. (1997). Biotechnology of Antibiotics.

[2] Aparicio J.F., Mendes M.V., Anton N., Recio E., Martin J.F. (2004). Polyene macrolide antibiotic biosynthesis. Curr. Med. Chem..

[3] Szlinder-Richert J., Mazerski J., Cybulska B., Grzybowska J., Borowski E. (2001). MFAME, *N*-methyl-*N*-d-fructosyl amphotericin B methyl ester, a new amphotericin B derivative of low toxicity: Relationship between self-association and effects on red blood cells. Biochim. Biophys. Acta Gen. Sub..

[4] Ogasawara Y., Katayama K., Minami A., Otsuka M., Eguchi T., Kakinuma K. (2006). Cloning, Sequencing, and Functional Analysis of the Biosynthetic Gene Cluster of Macrolactam Antibiotic Vicenistatin in Streptomyces halstedii. Chem. Biol..

[5] Cereghetti D.M., Carreira E.M. (2006). Amphotericin B: 50 Years of Chemistry and Biochemistry. Synthesis.

[6] Carmody M., Murphy B., Byrne B., Power P., Rai D., Rawlings B., Caffrey P. (2005). Biosynthesis of Amphotericin Derivatives Lacking Exocyclic Carboxyl Groups. J. Biol. Chem..

[7] Gantt R.W., Peltierpain P., Thorson J.S. (2011). Enzymatic methods for glyco (diversification/randomization) of drugs and small molecules. Nat. Prod. Rep..

[8] Zotchev S.B. (2003). Polyene Macrolide Antibiotics and their Applications in Human Therapy. Curr. Med. Chem..

[9] Baginski M., Czub J., Sternal K. (2010). Interaction of amphotericin B and its selected derivatives with membranes: Molecular modeling studies. Chem. Rec..

[10] Atta H.M., Selim S.M., Zayed M.S. (2012). Natamycin antibiotic produced by Streptomyces sp.: Fermentation, purification and biological activities. J. Ame. Sci..

[11] Stark J. (2003). Natamycin: An effective fungicide for food and beverages. Nat. Antimicrobials Minim. Process. Foods.

[12] Austin A., Lietman T., Rose-nussbaumer J. (2017). Update on the management of infectious keratitis. Ophthalmology.

[13] Priya A.B., Kalyan M. (2018). In vitro leishmanicidal effects of the anti-fungal drug natamycin are mediated through disruption of calcium homeostasis and mitochondrial dysfunction. Apoptosis.

[14] Te Welscher Y.M., Jones L., Van Leeuwen M.R., Dijksterhuis J., de Kruijff B., Eitzen G., Breukink E. (2010). Natamycin Inhibits Vacuole Fusion at the Priming Phase via a Specific Interaction with Ergosterol. Antimicrob. Agents Chemother..

[15] Tanner W. (2014). Membrane transport inhibition as mode of action of polyene antimycotics: Recent data supported by old ones. Food Technol. Biotechnol..

[16] Van Leeuwen M.R., Golovina E.A., Dijksterhuis J. (2009). The polyene antimycotics nystatin and filipin disrupt the plasma membrane, whereas natamycin inhibits endocytosis in germinating conidia of Penicillium discolor. J. Appl. Microbiol..

[17] Mccall L.I., Aroussi A.E., Choi J.Y., Vieira D.F., Muylder G.D., Johnston J.B., Chen S., Kellar D., Siqueira-Neto J.L., Roush W.R. (2015). Targeting Ergosterol Biosynthesis in Leishmania donovani: Essentiality of Sterol 14 alpha-demethylase. PLoS Negl. Trop. Dis..

[18] Dutta S., Whicher J.R., Hansen D.A., Hansen W.A., Chelmer J.A., Congdon G.R., Alison R.H.N., Kristina H., Sherman D.H., Smith J.L., Skiniotis G. (2014). Structure of a modular polyketide synthase. Nature.

[19] Skiba M.A., Sikkema A.P., Fiers W.D., Gerwick W.H., Sherman D.H., Aldrich C.C., Smith J.L. (2016). Domain Organization and Active Site Architecture of a Polyketide Synthase C-methyltransferase. ACS Chem. Biol..

[20] Curran S.C., Hagen A., Poust S., Chan L.J.G., Garabedian B.M., Rond T., Baluyot M.J., Vu J.T., Lau A.K., Yuzawa S. (2018). Probing the flexibility of an iterative modular polyketide synthase with non-native substrates in vitro. ACS Chem. Biol..

[21] Rittner A., Paithankar K.S., Vu K.H., Grininger M. (2018). Characterization of the polyspecific transferase of murine type I fatty acid synthase (FAS) and implications for polyketide synthase (PKS) engineering. ACS Chem. Biol..

[22] Ferscht A. (1985). Enzyme Structure and Mechanism.

[23] Kormana T.P., Crawfordb J.M., Labonteb J.W., Newmanb A.G., Wongc J., Townsendb C.A., Tsaia S.C. (2010). Structure and function of an iterative polyketide synthase thioesterase domain catalyzing Claisen cyclization in aflatoxin biosynthesis. Proc. Natl. Acad. Sci. USA.

[24] Chen X.P., Shi T., Wang X.L., Wang J.T., Chen Q.H., Bai L.Q., Zhao Y.L. (2016). Theoretical studies on the Mechanism of Thioesterase-catalyzed Macrocyclization in Erythromycin Biosynthesis. ACS Catal..

[25] Trauger J.W., Kohli R.M., Walsh C.T. (2001). Cyclization of Backbone-Substituted Peptides Catalyzed by the Thioesterase Domain from the Tyrocidine Nonribosomal Peptide Synthetase. Biochemistry.

[26] Parthasarathy S., Murthy M.R.N. (1997). Analysis of temperature factor distribution in high-resolution protein structures. Protein Sci..

[27] Pan X.Y., Shen H.B. (2009). Robust Prediction of B-Factor Profile from Sequence Using Two-Stage SVR Based on Random Forest Feature Selection. Protein Peptide Lett..

[28] Laskowski R.A., Swindells M.B. (2011). LigPlot+: Multiple ligand-protein interaction diagrams for drug discovery. J. Chem. Inf. Model..

[29] Koch A.A., Hansen D.A., Shende V.V., Furan L.R., Houk K.N., Gonzalo Jiménez-Osés G., Sherman. D.H. (2017). A Single Active Site Mutation in the Pikromycin Thioesterase Generates a More Effective Macrocyclization Catalyst. J. Am. Chem. Soc..

[30] Lu T., Chen F.W. (2012). Multiwfn: A multifunctional wavefunction analyzer. J. Comput. Chem..

[31] Szklarczyk D., Morris J.H., Cook H., Kuhn M., Wyder S., Simonovic M., Santos A., Doncheva N.T., Roth A., Bork P. (2017). The STRING database in 2017: Quality-controlled protein-protein association networks, made broadly accessible. Nucleic Acids Res..

[32] Onur S., Pemra O. (2018). gRINN: A tool for calculation of residue interaction energies and protein energy network analysis of molecular dynamics simulations. Nucleic Acids Res..

[33] Li J., Sun R., Wu Y.H., Song M.Z., Li J., Yang Q.Y., Chen X.Y., Bao J.K., Zhao Q. (2017). L1198F Mutation Resensitizes Crizotinib to ALK by Altering the Conformation of Inhibitor and ATP Binding Sites. Int. J. Mol. Sci..

[34] Lee J., Gokey T., Ting D., He Z.H., Guliaev A.B. (2018). Dimerization misalignment in human glutamate-oxaloacetate transaminase variants is the primary factor for PLP release. PLOS ONE.

[35] Liu W.P., Liu G.J., Zhou H.Y., Fang X., Fang Y., Wu J.H. (2016). Computer prediction of paratope on antithrombotic antibody 10B12 and epitope on platelet glycoprotein VI via molecular dynamics simulation. BioMed. Eng. OnLine.

[36] Shi T., Liu L.X., Tao W.T., Luo S.G., Fan S.B., Wang X.L., Bai L.Q., Zhao Y.L. (2018). Theoretical studies on the Catalytic Mechanism and Substrate Diversity for Macrocyclization of Pikromycin Thioesterase. ACS Catal..

[37] Giraldes J.W., Akey D.L., Kittendorf J.D., Sherman D.H., Smith J.L., Fecik R.A. (2006). Structural and mechanistic insights into polyketide macrolactonization from polyketide-based affinity labels. Nat. Chem. Biol..

[38] (2005). Accelrys Discovery Studio Visualizer 3.5.

[39] Laskowski R.A., MacArthur M.W., Moss D.S., Thornton J.M. (1993). PROCHECK: A program to check the stereochemical quality of protein structures. J. Appl. Cryst..

[40] (2014). Amber 2014.

[41] Jakalian A., Bush B.L., Jack D.B., Bayly C.I. (2000). Fast, efficient generation of high-quality atomic Charges. AM1-BCC model: I. Method. J. Comput. Chem..

[42] Jorgensen W.L., Chandrasekhar J., Madura J.D., Impey R.W., Klein M.L. (1983). Comparison of simple potential functions for simulating liquid water. J. Chem. Phys..

[43] (2009). Gaussian 09.

[44] Duan Y., Wu C., Chowdhury S., Lee M.C., Xiong G.M., Zhang W., Yang R., Cieplak P., Luo R., Lee T. (2003). A point-charge force field for molecular mechanics simulations of proteins based on condensed-phase quantum mechanical calculations. J. Comput. Chem..

[45] Ryckaert J.P., Ciccotti G., Berendsen H.J.C. (1977). Numerical integration of the Cartesian equations of motion of a system with constraints: Molecular dynamics of *N*-alkanes. J. Chem. Phys..

[46] Darden T., York D., Pedersen L. (1993). Particle mesh Ewald: An N.log(N) method for Ewald sums in large systems. J. Chem. Phys..

[47] Vreven T., Byun K.S., Komáromi I., Dapprich S., Montgomery J.A., Morokuma K., Frisch M.J. (2006). Combining quantum mechanics methods with molecular mechanics methods in ONIOM. J. Chem. Theory Comput..

[48] Vreven T., Frisch M., Kudin K., Schlegel H., Morokuma K. (2006). Geometry optimization with QM/MM methods II: Explicit quadratic coupling. Mol. Phys..

[49] Zhao Y., Truhlar D.G. (2008). The M06 suite of density functionals for main group thermochemistry, thermochemical kinetics, noncovalent interactions, excited states, and transition elements: Two new functionals and systematic testing of four M06-class functionals and 12 other functionals. Theor. Chem. Acc..

[50] Rassolov V.A., Ratner M.A., Pople J.A., Redfern P.C., Curtiss L.A. (2001). 6-31G* Basis Set for Third-Row Atoms. J. Comput. Chem..

[51] Swanson J.M.J., Henchman R.H., McCammon J.A. (2004). Revisiting free energy calculations: A theoretical connection to MM/PBSA and direct calculation of the association free energy. Biophys. J..

