# Supplementary material for: Insight into Structural Characteristics of Protein-Substrate Interaction in Pimaricin Thioesterase
Source: Int J Mol Sci. 2019 Feb 18;20(4):877. doi: 10.3390/ijms20040877 (PMC6412417; doi:10.3390/ijms20040877)
Supplement: Supplementary file 1 [file ijms-20-00877-s001.pdf]

International Journal of *Molecular Sciences*

Supporting Information

Insight into Structural Characteristics of Protein-Substrate Interaction in Pimaricin Thioesterase

Shuobing Fan [1], Rufan Wang [1], Chen Li [1], Linquan Bai [1], Yi-Lei Zhao [1], Ting Shi [1,*]

[1] State Key Laboratory of Microbial Metabolism, Joint International Research Laboratory of Metabolic and Developmental Sciences, School of Life Sciences and Biotechnology, Shanghai Jiao Tong University, Shanghai 200240, China

* Correspondence: tshi@sjtu.edu.cn; Tel/Fax: +86-21-34207347

Received: date; Accepted: date; Published: date

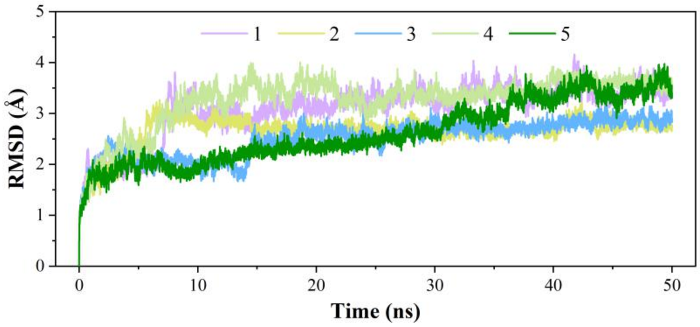

Figure S1. RMSD of five pima-TE simulations with the first frame as a reference.

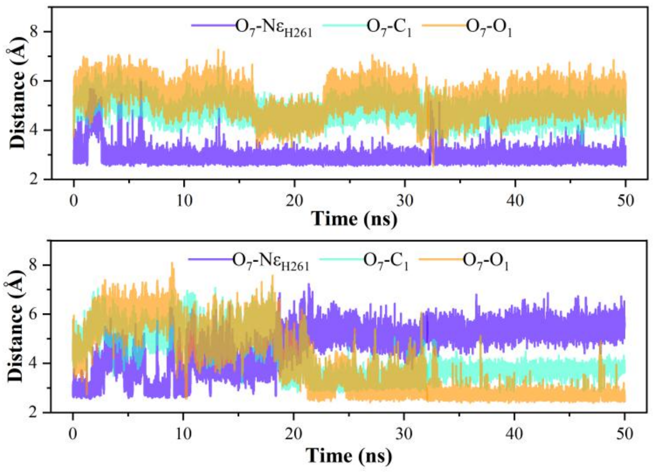

Figure S2. Distances O_7-$N\varepsilon_{H261}$, O_7-C_1 and O_7-O_1 in wild type trajectory md4 and md1 colored in purple, cyan and yellow, respectively.

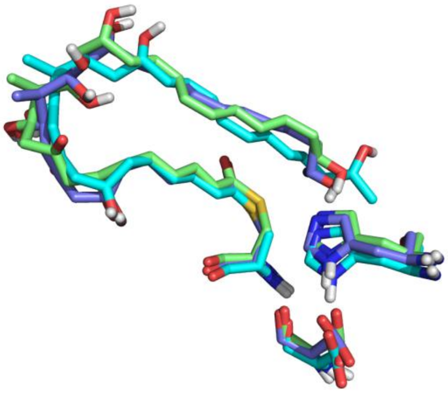

Figure S3. Dominant polyketide chain structure in S138C md1-3.

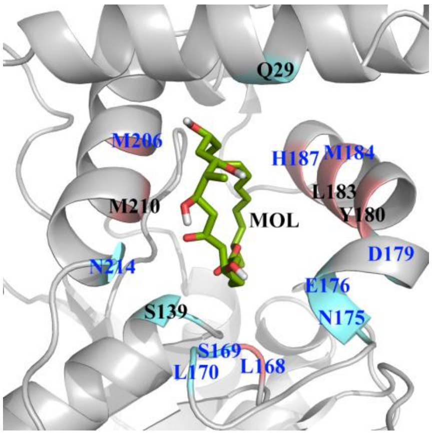

Figure S4. Positional distribution of residues contributing to VDW (light blue) or electrostatic (salmon) forces in protein-product interactions. Residues labeled in black indicated those identical as polyketide chain system, while newly-emerged ones labeled in blue.

Table S1. Proportion of PRS formation in wild type and mutated trajectories.

Category	Traj. No.	PRS Proportion	Average
wild type (50ns)	1	0.65%	4.37%
	2	0.06%	
	3	1.58%	
	4	18.80%	
	5	0.74%	
Q29A (30ns)	1	0.29%	0.54%
	2	1.19%	
	3	0.15%	
M210G (30ns)	1	0.11%	0.72%
	2	2.02%	
	3	0.03%	

R186F (30ns)	1	0.54%	1.64%
	2	1.71%	
	3	2.68%	
R186Y (30ns)	1	2.40%	18.14%
	2	10.19%	
	3	41.84%	
S138C (50ns)	1	1.76%	0.60%
	2	0.02%	
	3	0.02%	

Table S2. Proportion of hydrogen bond E80-R266 formation.

**Category**	**Traj. No.**	**Proportion of hydrogen bond (E80-R266)**	**Average Proportion**
wild type (50ns)	1	23.54%	35.71%
	2	6.28%	
	3	31.21%	
	4	50.31%	
	5	67.22%	
R186F (30ns)	1	49.65%	24.35%
	2	12.11%	
	3	11.29%	

Table S3. Proportion of hydrogen bond E80-R/Y186 formation.

**Category**	**Traj. No.**	**Proportion of hydrogen bond E80-R/Y186**
wild type (50ns)	1	6.59%
	2	4.72%
	3	57.72%
	4	56.54%
	5	49.60%
R186Y (30ns)	1	4.49%
	2	0.09%
	3	19.03%
